# The Identification and Interpretation of *cis*-Regulatory Noncoding Mutations in Cancer

**DOI:** 10.3390/ht8010001

**Published:** 2018-12-20

**Authors:** Minal B. Patel, Jun Wang

**Affiliations:** Centre for Molecular Oncology, Barts Cancer Institute, Queen Mary University of London, London EC1M 6BQ, UK; m.b.patel@qmul.ac.uk

**Keywords:** NCMs, *cis*-regulatory, high-throughput sequencing, computational analysis, cancer

## Abstract

In the need to characterise the genomic landscape of cancers and to establish novel biomarkers and therapeutic targets, studies have largely focused on the identification of driver mutations within the protein-coding gene regions, where the most pathogenic alterations are known to occur. However, the noncoding genome is significantly larger than its protein-coding counterpart, and evidence reveals that regulatory sequences also harbour functional mutations that significantly affect the regulation of genes and pathways implicated in cancer. Due to the sheer number of noncoding mutations (NCMs) and the limited knowledge of regulatory element functionality in cancer genomes, differentiating pathogenic mutations from background passenger noise is particularly challenging technically and computationally. Here we review various up-to-date high-throughput sequencing data/studies and in silico methods that can be employed to interrogate the noncoding genome. We aim to provide an overview of available data resources as well as computational and molecular techniques that can help and guide the search for functional NCMs in cancer genomes.

## 1. Introduction

Cancer is potentiated with the accumulation of mutations, some of which are inherited in the germline, but the vast majority arise in somatic cells [1]. These variations include single nucleotide substitutions, insertions and deletions (INDELS) and copy number alterations and translocations. Despite these changes, only a very small number of them are believed to be pathogenic (i.e., driver mutations), with the majority being passenger mutations (i.e., alterations not directly implicated in tumour development). The identification of driver mutations in genes is essential in unravelling key molecular events that occur in cancer cells, and also providing candidate biomarkers for therapeutic intervention [2]. A huge body of whole-exome sequencing (WES) projects such as The Cancer Genome Atlas (TCGA), which capture the exon coding regions of the genome, have substantially advanced the understanding of coding mutations in cancer, with key driver genes and mutations established across many cancer types. This has led to a wave of targeted precision medicine in various cancers, including chronic myelogenous leukaemia [3], breast [4], lung cancers [5,6] and melanomas [7,8,9].

However, coding sequences make up less than 2% of the human genome, with the other 98% comprising noncoding DNA. Our understanding of noncoding mutations (NCMs) and their functional consequences in cancer development and progression is still very limited mainly due to the lack of effective tools to study them. The recent emergence of comprehensive regulatory annotation from the Encyclopedia of DNA Elements (ENCODE) project [10], Roadmap Epigenomics Consortium [11] and the FANTOM5 project [12] have revolutionised our understanding of noncoding sequences, providing powerful resources for annotating noncoding regulatory elements and variations across tissue and cell types. The availability of large-scale whole genome sequencing (WGS) projects, such as those by the International Cancer Genome Consortium (ICGC), and other noncoding data sets such as chromatin immunoprecipitation and sequencing techniques (ChIP-seq) and noncoding RNA sequencing (RNA-seq), has further provided a plethora of genomic data and noncoding elements across cancer types, allowing for in-depth investigation and systematic search for functional NCMs. There is much evidence to suggest that recurrent mutations within the noncoding elements are functionally important [2]. To identify those noncoding drivers will undoubtfully further enrich our understanding of molecular pathogenesis of many cancers and provide novel targets for diagnostics and therapeutics.

Amongst the effort of searching for functional NCMs, there have been many studies that have employed and integrated various regulatory annotation and genome-scale sequencing tools across cancer types. Many computational algorithms and pipelines have also been developed to perform the data analyses and identify/prioritise functional NCMs. Here we review recent high-throughput studies and technologies used to study NCMs in cancer.

## 2. Regulatory Regions of the Noncoding Genome and Functional Effects

**Figure 1:**
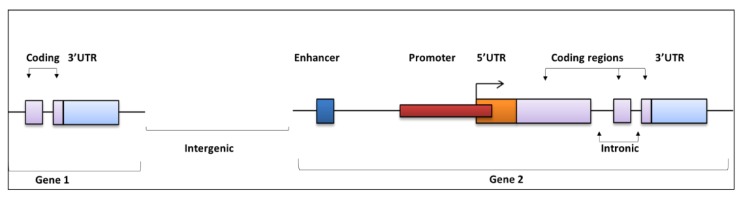
The distribution of regulatory and coding regions along a gene. Driver mutations can be found in both coding and noncoding regions. Mutations within the enhancer regions (dark blue) may create binding motifs for regulatory factors that can promote or inhibit gene expression. Similarly, mutations within the promoter regions can affect binding sites that regulate transcription. Coding mutations (within purple region) can have many functional affects. For example, the alteration of amino acids can disrupt protein folding. Mutations within UTR regions can have numerous affects, such as disrupting miRNA targeting. Moreover, mutations located within intronic regions are likely to affect splicing and gene expression, whilst mutations located in intergenic regions may affect genes up- or downstream of their location.

Noncoding regions can be broadly split into *cis*-regulatory regions and noncoding RNAs (ncRNA) [2]. *Cis*-regulatory regions comprise promoters and distal elements (promoters, enhancers and insulators) (Figure 1), and regulate transcriptional activity and complex spatial and temporal gene expression following the binding of transcription factors [2,13]. NcRNAs comprise microRNAs (miRNAs), other small noncoding RNA species and long-noncoding RNAs (lncRNA, >200 bp), which regulate the stability, post-transcription or translation of protein-coding genes [14,15]. NCMs can occur in any part of these regions and have been identified throughout the genome. Untranslated regions (5’ and 3’) are also an important class of regulatory elements that harbour driver mutations implicated in tumourigenesis [16,17,18,19,20]. 5’ UTR regions play a significant role in controlling translation initiation, thus important mutations here can impact the initiation complex and expression downstream. 3’ UTR regions comprise binding sites for regulatory proteins and miRNAs. miRNA binding decreases gene expression by inhibiting translation or degradation of the transcript, thus disruption of these binding sites can lead to oncogenic expression [14]. Mutations within intronic regions can also have fundamental implications in tumourigenesis, directly affecting splicing events and leading to malignant transcript isoforms [21].

NCMs also reside in intergenic regions. Weinhold et al. previously reported that intergenic regions harboured the highest mutational burden in a WGS study of 863 tumour genomes [22]. Mutations residing here can impact genes locally and distally, but can be difficult to functionally interpret [23]. NCMs in intergenic and other regions can also affect gene regulatory factors including the epigenetic changes involved in chromatin conformation, DNA accessibility and acetylation and methylation of N-terminal histone tails [24]. Trans-regulatory regions are also of noteworthy importance as they encode for transcription factors which bind to and regulate the activity of *cis*-elements [25]. In this review we primarily focus on NCMs in *cis*-regulatory regions, particularly promoters and enhancers, and various sequencing and computational techniques implemented to identify them.

### Mode of Action for NCMs

**Figure 2:**
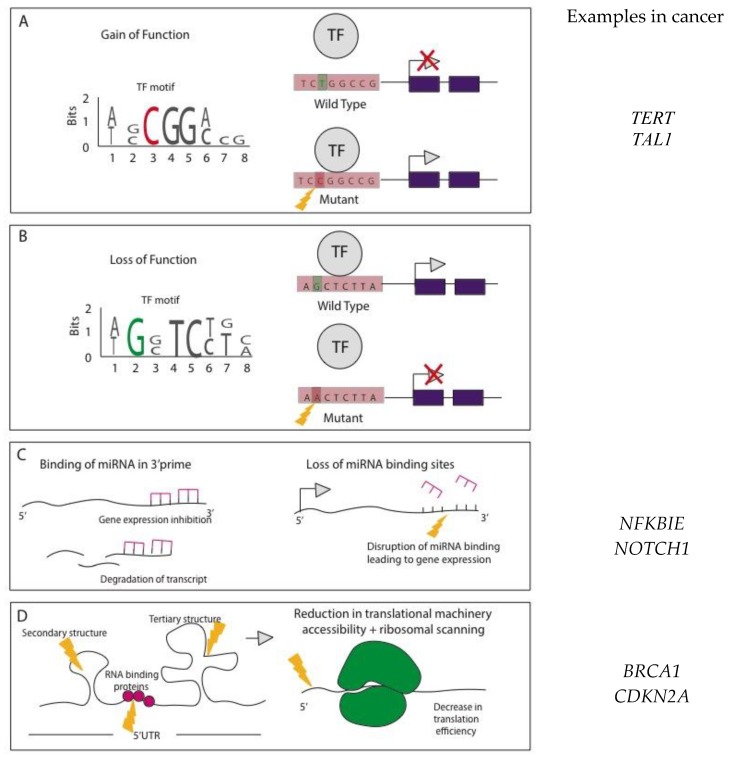
Functional effects of noncoding mutations (NCMs). (**A**) Mutations within promoter (e.g., *TERT*) and enhancer regions (e.g., *TAL1)* can create transcription factor (TF) binding motifs in a gain-of-function manner allowing the binding of transcriptional activators leading to oncogenic transcription and gene expression [26,27,28]. (**B**) Alternatively, mutations within regulatory regions can create the loss of transcription factor binding sites, leading to transcriptional repression. (**C**) miRNA binding within the 3’ UTR control gene expression, by inhibiting translation or marking transcripts for degradation. Mutations that disrupt these binding sites can lead to oncogenic expression (e.g., *NFKBIE* and *NOTCH1* genes) [16,17]. (**D**) Mutations within the 5’ UTR can alter the secondary and tertiary structures, as well as trans-acting RNA binding protein sites. These alterations can affect translation efficiency and mRNA stability (such as observed in *BRCA1* and *CDKN2A* genes) [18,29].

The mode of action of NCMs is incredibly complex. In addition to the alterations described above, NCMs can be broadly classified into gain and loss of function. In promoter and enhancer regions gain-of-function mutations result in the creation of TF-binding sites, which can lead to downstream oncogenic transcriptional activity as previously reported in both promoter [26] and enhancer regions [27,28] (Figure 2A). Loss of function mutations results in the loss of TF-binding sites leading to transcriptional inhibition of downstream genes. We summarise some of the functional effects of NCMs in Figure 2.

## 3. Noncoding Genomic Variations and Mutations Identified across Large-Scale Cancer Studies

**Figure 3:**
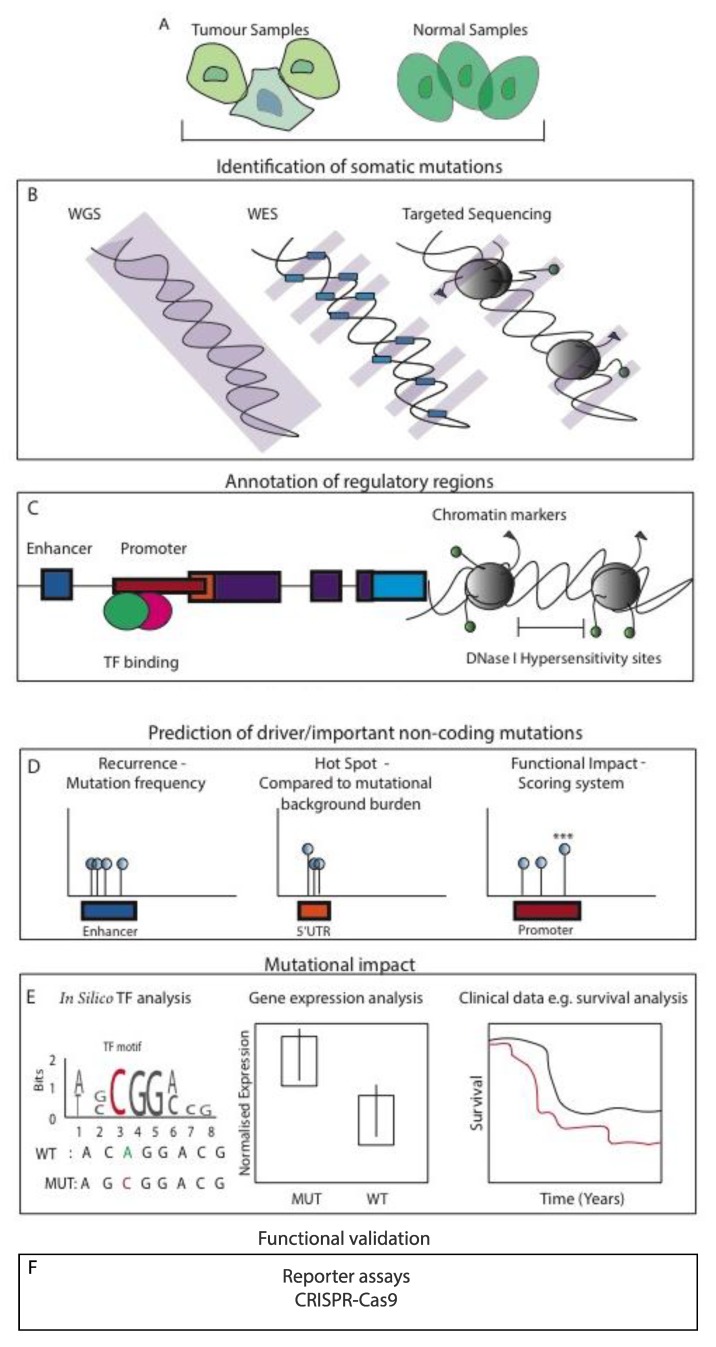
Study designs commonly used to identify functional NCMs in cancer. (**A**) Cells are generally taken from tumour and matched normal samples (biopsy, surgical resection and blood). (**B**) Various high-throughput techniques are used to identify somatic mutations. Whole-genome sequencing (WGS) enables the identification of common and rare variants genome-wide. Whole-exome sequencing (WES) can be used to identify mutations within exon coding regions, or more targeted methods such as chromatin immunoprecipitation sequencing (ChIP-seq) can be utilised to identify variants within regulatory regions. (**C**) Somatic mutations identified from high-throughput techniques can be further annotated and filtered depending on their regulatory location using data repositories such as ENCODE. (**D**) Computational algorithms are used to predict and filter potential driver/important mutations. Recurrent mutational analysis identifies regulatory regions that are enriched for mutations across the region in many patients. Hotspot (or cluster) analysis looks for mutations located within close proximity to each other. Functional scoring analysis uses a combination of annotation methods to score putative functional mutations providing significance values for each mutation. (**E**) Studies also integrate other layers of information, such as the use of in silico transcription factor (TF) repositories to identify the gain or loss of TF binding motifs. Matching expression data enables the analysis of mutant effects in patients on proximal gene expression compared to wild type (WT). Furthermore, corresponding clinical data enables the interrogation of survival based on mutation presence and gene expression. (**F**) Variants that pass these steps generally undergo reporter assays/CRISPR-Cas9-based functional validation to further determine the biological significance.

Many high-throughput platforms have been used to uncover important mutations within the noncoding genome of many cancers. Whole genome sequencing (WGS) has been used to comprehensively study a plethora of genomic alterations, elucidating the whole mutational landscape of cancer. WGS approaches can be additionally integrated with more targeted methods to help guide the interpretation of the simple somatic mutation (SSM) data, such as the use of WES and targeted sequencing to study mutations within/near promoter regions. Moreover, ChIP-seq can be incorporated to capture enhancer regulatory regions and transcription factor (TF) binding sites. Further epigenome-centric approaches comprise the use of chromosome conformation capture technologies Chromatin Interaction Analysis Paired End-Tag Sequencing (ChIA-PET) and Hi-C, which demonstrate the 3D organisation of the genome in high-resolution uncovering the true chromatin interactions with their target genes [30,31]. Importantly, many studies incorporate the use of matched expression data, to explore the impact of *cis*-regulatory mutations on proximal located coding genes, thus adding an informative layer to increase the detection of mutation functional significance. Also, RNA-seq data can be used to infer genes with allele imbalance (AI), further providing evidence that potential *cis*-acting genomic lesions have occurred within the regulatory sequences of AI targeted genes. We summarise the most commonly used study designs for the identification of functional NCMs in Figure 3. Various noncoding mutation studies are also summarised and listed in Table 1.

### 3.1. Whole-Genome Centric Approaches

#### 3.1.1. Whole-Genome Scans Using WGS

The most common method of scanning the whole genome for important NCMs is the use of ‘hotspot’ (or cluster) analysis, which is a method of identifying genomic loci enriched for mutations within short distances of each other, in comparison to the background mutational burden (masking coding regions). This reduction in dimensionality increases the statistical power with the rise in mutation frequency per sequence window [30]. In a small genome-wide study, Hu et al., used WGS data from 31 Chinese children with T cell acute lymphoblastic leukaemia (T-ALL) [32]. They implemented three systematic approaches. First, a hotspot method was implemented to identify highly mutated genomic loci within 21 base pairs (bp). Second, annotated regulatory regions derived from Ensemble resources were searched for mutation enrichment. This restrictive method increased the power to potentially detect NCMs with functional attributes. Lastly, TF binding sites overlapping mutations were analysed to identify the gain or loss of TFs between mutant and WT regions. By doing so, recurrent NCMs within T-ALL oncogenes *LMO1*, *LMO2* and *TAL1*, were identified. Also, *LMO1* and *TAL1* were significantly associated with increases in gene expression changes, with insertions nearby of *TAL1* and *LMO1* creating *MYB* binding sites in a number of patients [32].

#### 3.1.2. Recurrently Mutated Noncoding Clusters

A Recent study has utilised WGS data to identify NCMs within aberrant somatic hypermutation (aSHM) regions of genes, caused by the enzyme activation-induced cytidine deaminase (AID). AID is encoded by the gene *AIDA*, and induces mutations, changing a C:G match to a U:G mismatch and is implicated in many lymphoid cancers particularly in the development of B cell lymphomas [17]. Using diffuse large B cell lymphoma (DLBCL) tumour–normal matched sample pairs from 153 patients, Arthur et al., incorporated the use of two algorithms to identify NCMs within these aSHM regions [17]. The first algorithm identified regions of enriched SSM density in comparison to the background (excluding coding regions) and the second inferred the presence of peak regions of elevated local mutation rates. This combined strategy identified recurrent mutations in the 3’ UTR region of the *NFKBIZ* gene and was further validated in 13.9% of 338 additional DLBCL cases with targeted sequencing. In addition, within these 338 cases, Arthur et al., also used matching RNA-seq raw data reads to infer allelic imbalance (AI), using Samtools ‘mpileup’ [33] to quantify the number of reads supporting the reference and alternative allele for each variant. AI in *NFKBIZ* was identified towards the mutant allele, and further experimentally validated using droplet digital polymerase chain reaction (ddPCR) in two cell lines [17]. 

Pan-cancer studies provide an effective strategy to integrate cancer genome data and identify common NCMs and regulatory elements across different cancer subtypes. For example, Melton et al., profiled WGS data from 436 patients across eight cancers [34]. Focusing on the DNase I Hypersensitivity sites (DHS) or TF binding peaks from RegulomeDB resources [35], they identified eight recurrently mutated genomic loci in proximity to cancer-associated genes, such as *GNAS*, *INPP4B* and *MAP2K2*, following a statistical enrichment model. However, the regulatory impact of these eight regions was not validated; therefore their regulatory implications have yet to be deciphered. Consistent with earlier pan-cancer studies [22,36], mutational hotspots were also identified near *TERT* and *PLEKHS1* genes in this study. More recently, a pan-cancer study by Zhang et al., [20] studied the functional consequences of NCMs within 930 tumour–normal matched whole genomes across 22 cancers with the integration of transcriptomics and transcriptional interaction maps. To identify recurrently mutated loci, they used a hotspot analysis to search for mutations within 50 bp of one another genome-wide, identifying 193 somatic expression quantitative trait loci (eQTLs) regulating 196 genes. Three of which were experimentally validated (*DAAM1*, *MTG2* and *HYI*). Furthermore, Zhang et al., studied the convergence of these noncoding mutations and previously documented coding mutations on network pathways. Aggregating all affected genes and analysing with the Network-Based Stratification algorithm (a method of integrating somatic cancer genomes with gene networks) [37] they identified four subtypes of interest.

### 3.2. Targeted and Integrative Approaches

#### 3.2.1. Promoter-Centric

Thus far, the most notable example of noncoding variations in cancer is from the identification of driver mutations in the promoter regions of the telomerase reverse transcriptase (*TERT*) gene. *TERT* promoter mutations were first described in melanoma [38,39]. Since then they have also been described in gliomas and a subset of tumours in tissues with low rates of self-renewal, such as hepatocellular and urothelial carcinomas [40]. Importantly, *TERT* has been clinically correlated with poor survival in clear-cell renal carcinomas [26], gliomas [41], bladder [42] and thyroid cancers [36], demonstrating the potential of NCMs as clinical biomarkers and therapeutic targets [43].

In a recent melanoma study, Shain et al., implemented a combination of low depth WGS (13×) and WES (89×) sequencing of 20 desmoplastic melanomas with matched normal DNA. Integrating these high-throughput techniques they identified recurrent promoter mutations in *NFKBIE* [43]. This was further validated by targeted sequencing (216×) of 293 genes in 42 neoplastic and non-neoplastic formalin-fixed paraffin-embedded primary desmoplastic melanomas [44].

Rheinbay et al., 2017 developed an adapted exome assay to capture not only the exome, but also the promoter elements and additional regulatory elements such as enhancer regions. This was followed by next generation sequencing at a median depth of 80× in 360 primary breast tumours and corresponding normal counterparts. They identified mutations in breast cancers within regions of high alipoprotein B messenger RNA-editing enzyme catalytic (APOBEC), a region of conserved cytidine deaminases and a large source of mutations in cancer [45,46]. A SignatureAnalyser tool [47] was firstly used to remove these mutations with high APOBEC probability from their analysis. Next, to identify recurrent mutations, Rheinbay et al., developed an analytical pipeline (MutSigNC), which takes into consideration patient-specific mutation rates, sequence coverage and mutation clustering. This pipeline then compared this mutation data to the background variant burden of other promoter regions, taking into consideration factors such as GC-rich sequences and chromatin states which specifically affect promoter elements [46]. Nine genes with promoter associated mutations were identified, three of which (*FOXA1*, *RMP* and *NEAT1*) were significantly associated with gene activity in luciferase reporter assay experiments. They further confirmed 97% of mutations in promoter regions by deeply resequencing targeted regions in 47 patients, with at least 1,000× coverage.

Similarly, Nik-Zainal et al., used whole-genome data from 560 breast cancers and normal counterparts to identify recurrent mutations in coding and noncoding genomes [45]. The noncoding analysis involved the partitioning of the genome into separate regulatory elements and gene features. Elements were then analysed independently for mutation rates using a negative binominal regression approach to determine the variation of mutations across each element compared to the background distributions. Paradoxically to later studies [46], APOBEC regions were not removed from the initial analysis, subsequently recurrent mutations were identified within the APOBEC regions in the promoter of *PLEKHS1* and *TBC1D12*. Nik-Zainal et al., suggested that these mutated loci were therefore regions of hypermutability rather than driver mutations [45].

Feigin et al., described a Genomic Enrichment Computational Clustering Operation (GECCO) to uncover recurrent regulatory mutations in the *cis*-regulatory regions of 308 pancreatic ductal adenocarcinoma PDAC patient with WGS data and matched expression data [43]. To firstly filter WGS data, the algorithm tool Funseq2 was utilised [48]. This uses a weighted scoring system to filter and prioritise somatic NCMs. Subsequent mutations were further filtered to keep those mutations, with the most likely functional impact, overlapping TF binding peak annotations obtained from ENCODE ChIP-seq experiments in 72 cancer cell lines [10]. Using permutation testing, GECCO calculates the mutation rate in each regulatory region and associates with proximal gene expression and pathway analysis. A total of 16 genes with significant NCMs in PDAC patients were identified. Pathway analysis of these genes, using The Database for Annotation, Visualisation and Integrated Discovery (DAVID) resources [49], uncovered currently known PDAC pathways such as cell adhesion and Wnt signalling [43]. Furthermore, Feigin et al., used patient-matched expression-array data and identify low expression levels of the Protein Tyrosine Phosphatase Receptor Type N2 (*PTPRN2*) gene, which is also associated with poor overall survival [43]. However, the majority of the significantly mutated regulatory regions (*PTPRN2* not included in significantly mutated list) were not associated with gene expression changes, suggesting possible other roles yet to be established for these genomic loci.

#### 3.2.2. Active Enhancer Centric

NCMs have been observed in regions other than promoters, for example in T-ALL. A heterozygous 12 bp insertion was found in an intergenic region, creating a MYB-TF-binding site that results in a super-enhancer upstream of the *TAL1* oncogene [27]. Here, Mansour and colleagues used high-throughput techniques to mark increased acetylation of the histone H3 lysine 27 (H3K27ac)—a form of epigenetic change—which differentiates active enhancers from inactive/poised enhancer elements and promoters [50]. These protein-DNA interactions can be identified on a genome-wide scale using ChIP techniques, followed by sequencing of the underlying DNA (ChIP-seq) [27,51]. Sequencing of this enriched DNA allows the identification of potential enhancer associated sequence variants, using variant calling packages such as Samtools [52], Varscan2 [53] and MuTect2 [54], and customised pipelines to further filter out low quality variants. Mansour et al., identified a 12b insertion near the TAL1 oncogene creating an active enhancer with open chromatin conformation *de novo*. This enables transcription factors (TFs) to access and bind enhancer DNA motifs and regulate transcription [55]. Using in silico TF binding resources (UniProbe) [56] of previous experiments, they were able to predict a MYB binding site overlapping the insertion. This was further experimentally tested using MYB antibody ChIP-seq, validating the binding of MYB monoallelically to the mutant allele and driving *TAL1* expression [27].

Using similar techniques, Mansour and colleagues integrated a combination of ChIP-seq (H3K27ac marks) to enrich enhancer DNA with bioinformatics in 102 tumour cells [51]. Abraham et al., developed their own pipeline to identify short enhancer associated insertions using multiple alignment approaches in the underlying enriched DNA. Focusing on the functional significance of an 8-bp heterozygous insertion at the *LMO2* locus in the T-ALL cell line MOLT4, they identified TF binding and enhancer activity, driving heterozygous expression of *LMO2*. These findings were further reported in two more T-ALL cell lines, six paediatric and nine adult T-ALL patients [57]. Focusing on enhancer regions significantly reduces the noncoding genome search space and enables the identification of noncoding variants with potential functional activity at the gene control level [51].

In another study, using a combination of WGS and WES data, mutations in enhancer regions near the *PAX5* gene, a transcription factor implicated in B cell differentiation, was identified in chronic lymphocytic leukaemia (CLL) patients and diffuse large B cell, follicular and mantle-cell lymphomas [16]. In addition, Puente et al., used circularised chromosome conformation capture sequencing (4C-seq), a high through-put technique looking at genome-wide DNA contacts with a single genomic locus of interest [31]. This technique revealed the 3-dimensional (3D) interaction frequencies of the *PAX5* enhancer with the surrounding regions and demonstrated that the *PAX5* enhancer has contact with regions up to 330kb away [16]. To further confirm the transcriptional regulation abilities of this mutated enhancer region on nearby genes, Puente et al., interrogated RNA-seq data of genes located within 1Mb of the enhancer region. This demonstrated significantly increased expression of the *PAX5* gene only out of all 15 nearby genes, suggesting NCMs within the enhancer region transcriptionally regulate *PAX5* [16]. Utilising additional powerful high-throughput methods such as Hi-C, would overcome this method by facilitating the direct identification of NCMs and their target genes on a genome-wide scale [21]. However, it is sometimes unfeasible and expensive to experimentally validate all predicted interactions [58].

#### 3.2.3. Genome-Wide Chromosome Conformation

Most recently, Orlando et al., employed Hi-C data to decipher the spatial organisation of chromosomes and the regulation of NCMs in *cis*-regulatory elements on target gene expression. Hi-C on 19,023 promoter fragments in colorectal cancer cell lines was used alongside WGS data [59]. To identify functional NCMs in *cis*-regulatory elements, they analysed the transcriptional effects of NCMs identified in regulatory regions with Hi-C and matching RNA-seq data. Interactions were minimised to 1Mb from TSSs. By doing so they uncovered a recurrently mutated regulatory element interacting with the *ETV1* promoter. Matched gene expression data identified transcription upregulation, also correlating with poor survival in colorectal patients [59]. In an earlier study, using a combination of high-throughput techniques, Koues et al., investigated the transformation of normal germinal centre B cells to malignant follicular lymphoma (FL) B cells using an epigenome-centric approach [60]. A combination of formaldehyde assisted isolation of regulatory elements FAIRE-seq: a technique used to identify DNA segments that actively regulate transcription, ChIP-seq enrichment of active enhancers, and expression data, uncovered enhancers enriched with somatic mutations which disrupt TF-binding and subsequently target gene expression changes [60].

ChIA-PET is a high-throughput combination technique which incorporates both a chromatin immunoprecipitation technique and a chromosome capture (3C) technology, allowing for the analysis of both protein-DNA complexes and long-range interactions, genome-wide [21,61]. In a large-scale project investigating 300 liver cancers in a Japanese population, Fujimoto et al., used annotation resources from ENCODE to identify highly mutated regions overlapping DHS and ChIP-seq TF-binding sites [62], which uncovered mutations within four CCCTC-binding factor (CTCF) regions. ChIA-PET was then used and validated one of these CTCF-binding regions as an enhancer region located upstream of the *PRKCA* gene and downstream of *APOH* [62]. NCMs within this enhancer were significantly correlated with gene expression changes and luciferase reporter activity [62].

Outcomes from the use of high-throughput techniques are continuously expanding the catalogue of candidate NCMs. The use of integrated and more targeted approaches also greatly narrows down the search space to the most likely functional regions of the noncoding genome and increases statistical power in the identification of important NCMs. Despite this, the use of high-throughput techniques did not come without caveats.

## 4. High-Throughput Methods and Underlying Challenges

Discovering functional or driver mutations, especially in the noncoding genome where recurrent mutations are at a lower frequency, requires high-throughput technology with deep sequencing coverage, paired-end reads and large cohorts to establish statistical significance, contributing to the expense of studies [30]. This is particularly true for WGS, as the accuracy of mutation calling primarily relies on sequencing depth [63]. As previously mentioned, this can be overcome by high-depth targeted sequencing of regulatory regions of interest [46]. The alignment of WGS data to reference genomes can prove troublesome, as the human genome is riddled with repetitive and redundant regions. Thus, aligning short reads (usually 75–150 bp) to the whole genome accurately is a computationally extensive and difficult task with a large amount of alignment uncertainties and errors often occurring, which can lead to mutation calling faults [64,65,66]. Furthermore, accurately identifying somatic variants and rearrangements using WGS remains an open challenge facing the cancer bioinformatics community, as recent studies indicate that existing approaches overlap only ~20% [67]. With all this in mind, the ICGC-TCGA DREAM Genomic Mutation Calling Challenge has been initiated to identify the most accurate mutation detection algorithms, and establish state-of-the-art analytical pipelines [66,68]. It is therefore recommended that using an ensemble of pipelines or consensus calls of multiple algorithms can greatly improve mutation detection accuracy [65,69]. We summarise high-throughput technologies and their associated pros and caveats in Table 2.

ChIP-seq techniques not only provide a global snap-shot of chromatin accessibility, TF binding and histone remodelling modifications, to study the regulation of gene expression, it also provides a targeted approach to identify NCMs within putative functional regulatory regions. However, such techniques require large amounts of tissue to produce purified cells, which for some cancers such as those in the pancreas, are challenging to obtain surgically due to the asymptomatic progression of the disease. Thus, patients tend to present with inoperable disease. Also, some cancerous tissues like in the pancreas usually have low levels of tumour cellularity, with the presence of a large amount of non-tumour cells such as stromal and immune cells. With the high cell mortality rate as well as DNA degradation during the sample pre-processing stage, it is often very challenging to obtain enough purified cells and tumour DNA for ChIP [71]. Thus, organoid models are required to make sure enough tumour cells are available, adding another layer of complexity for ChIP-seq studies, especially for many solid cancers. Experimentally, ChIP-seq is more technically challenging in comparison to DNA methylation assays and RNA-seq for example, and as a consequence of this, the subsequent raw ChIP-reads require substantial quality control due to frequent poor quality [24]. Moreover, the peak signals are often quite noisy, requiring further recalibration and careful interpretation. Most ChIP-seq data available to date is cell line-specific, such as those provided by ENCODE and Epigenome Roadmap. From our own ongoing analysis, we have found that mutations called in cell lines often do not correspond to patient somatic mutations from WGS data. This is likely due to the disparity between histone modifications in cell line cultures, which can alter with media changes and increases in cell passaging comparative to in vivo settings [70,72].

Allele specific imbalance is an effective approach to detect the effect of genetic variation on gene expression in individual genomes [80]. This is typically achieved using raw RNA-seq reads to quantify reads in the reference and alternative allele and infer RNA genotypes of heterozygous SNPs. AI can then be used to locate *cis*-acting variants genome-wide and correlate with gene expression changes. Allele specific approaches are a powerful method of functionally annotating individual genomes, in particular for identifying rare *cis*-regulatory variants on a large scale [81]. However, methods are sensitive to technical issues with the processing of RNA-seq data such as thresholding, read depth/mapping and variant calling methods [77,78,82]. Furthermore, to detect AI at low frequency requires sufficient high read coverage, which for standard RNA-seq experiment (30–60 M reads) is limited. AI can also be inferred using targeted high-throughput techniques such as ChIP-seq and DNase-seq methods. Data sets from all technique approaches in the same cell line or individual can be combined to increase statistical power in the detection of AI [82].

**Figure 4:**
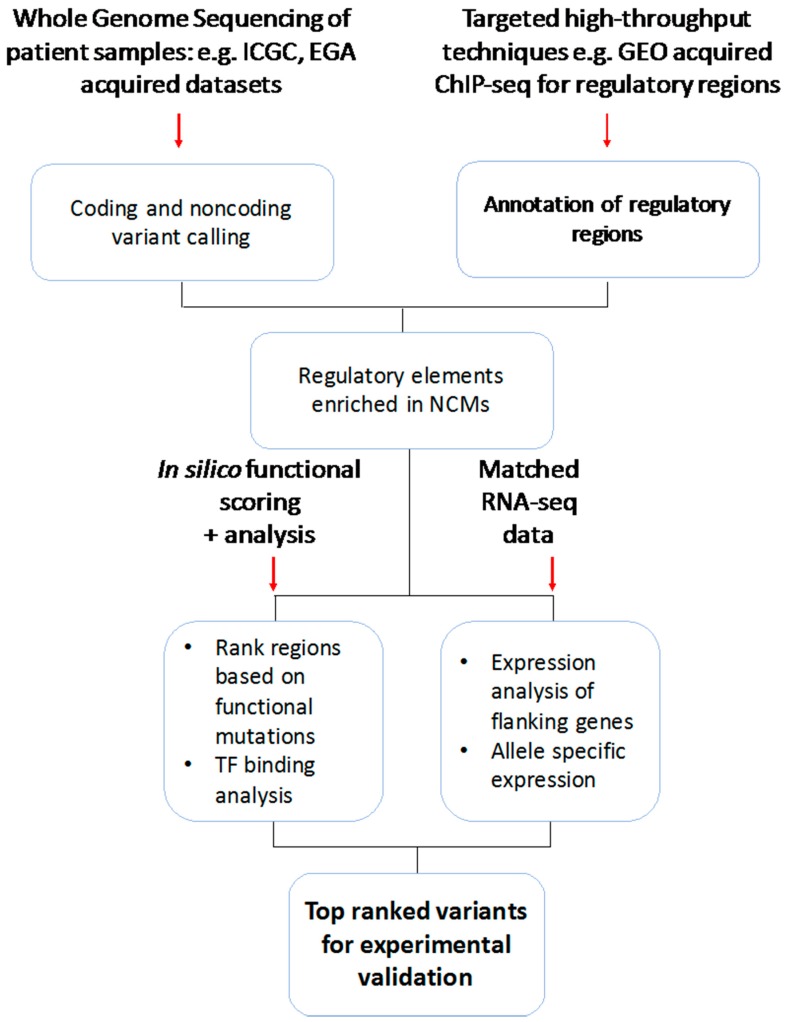
Integration of high-throughput data flowchart. This uses WGS data for SSM mutation data parallel to more targeted methods such as ChIP-seq to enrich the active enhancer regulatory regions for example. ChIP-seq data is used to guide the interpretation of WGS SSM data, identifying mutations within regions with putative regulatory effects. In silico functional scoring methods such as IW-scoring can then be used to annotate and rank mutations by their putative functional importance. TF repositories can also be utilised for de novo binding motifs overlapping mutated regions. In parallel matched RNA-seq data can be used to analyse proximal gene expression and allele specific expression. Together this should provide a list of top candidate variants to follow up with functional validation techniques. European Genome-Phenome Archive (EGA), Gene Expression Omnibus (GEO).

We believe the best practise is to integrate WGS with other high-throughput technologies such as WES, targeted sequencing, expression data (RNA-seq and expression arrays), epigenetic markers (ChIP-seq) and chromosome spatial organisation (chromosome capture technologies) to guide the use of WGS mutation data and narrow down the search space to regions of most functional impact [51], followed by functional validation in cell lines. Here we suggest an integrative workflow to identify functional NCMs based on multiomics data, shown in Figure 4.

## 5. Computational Resources and Techniques

Due to the sheer number of NCMs identified in cancer genomes, computational algorithms are often needed to annotate and score them first to select those that are most likely to be functional or deleterious for downstream analyses. To systematically study these noncoding variants, careful annotation is required, by determining the regulatory regions they map to and nearby/overlapping genes. These regulatory features usually include TF binding sites, open chromatin, various histone marked regions and TSS sites defined by ENCODE, Epigenome Roadmap and FANTOM5. Several online tools have been developed to help with gene and regulatory annotation for NCMs. For example, IW-scoring [83] developed annotation modules to provide information of all related regulatory regions and nearby genes for queried NCMs. It also integrates Ensembl Regulatory Build annotation [84] allowing for overlapping NCMs with predicted promoters and enhancers. Similarly, RegulomeDB [35] uses data from various regulatory resources of ENCODE, along with TF ChIP-seq data from the NCBI Sequence Read Archive [85], a large collection of eQTL data, as well as TF binding prediction by DNase footprints and positional weight matrices (PWMs), such as TRANSFAC [86], JASPAR [87] and UniPROBE [56], providing a comprehensive integrated approach to annotate regulatory variants (Table 3). In the last few years many computational algorithms have been developed to predict the functional consequences of noncoding variants. These methods often integrated available noncoding annotation features mentioned above to produce a continuous or discrete score for each variant in order to measure the likely functional impact of noncoding variants (shown in Table 3).

However, the computational techniques employed to generate the scores varied. For example, CADD [88], GWAVA [89], FATHMM-MKL [90] and Genomiser [91] use machine-learning algorithms to develop classifiers integrating a range of annotations such as regulatory features, conservation metrics, genic context and genome-wide properties to differentiate functional/deleterious from non-functional/benign variants. Other methods such as DeepSEA [92] and DeltaSVM [93] directly learned regulatory sequence motifs from ENCODEs large-scale chromatin profiling data, to enable predictions of chromatin effects for variants. FitCons [94] and LINSIGHT [95] estimated the selective pressure on the basis of patterns of polymorphism and divergence, and scored variants based on the likelihood of deleterious fitness consequences. FunSeq2 [48], Eigen [96] and IW-Scoring developed weighted scoring approaches to combine the relative importance of various annotation features to distinguish functional from non-functional variants [83]. RegulomeDB, on the other hand, employed a heuristic scoring system based on functional confidence of a variant, with increased confidence for variants located within functional locations [35]. The performances of these methods often vary when different sets of variants with distinct features are scored, thus similar to somatic variant calling strategies mentioned above, an ensemble approach or using a rank or consensus call of multiple methods become a powerful approach summarising multiple predictive evidences, hence increasing specificity and outperforming a single method. IW-Scoring is the first web portal to provide scores of most available methods and to generate an ‘ensemble-like’ score with weights, demonstrating stable performances and ranked consistently among the best performing methods for a diverse set of noncoding variants tested [83].

Independent of these functional scoring and prediction methods, approaches can be employed to assess whether a mutation or set of mutations have been observed at a higher frequency than expected, also comparing to background mutation rates. The most effective approach is to consider mutational heterogeneity [30,46]. The algorithm MutSigNC [46] for example, identifies recurrently mutated promoters by taking into consideration patient specific mutation rates, replication timing and patient-specific sequencing coverage when looking for mutation rates above expectation [46]. Similarly, LARVA [97] incorporates background models by integrating a comprehensive set of noncoding functional elements based on DHS sites and histone marks, whilst also considering replication rates to increase the mutation rate accuracy [97]. Other methods have been developed to infer positive selection, such as OncoDriveFML [98]. This algorithm analyses the pattern of somatic mutations bias across tumours and estimates the accumulated functional impact in genomic region of interest, compared to that expected by chance for the same number of mutations.

Noncoding mutations can also be further prioritised in regulatory elements with the identification of TF binding sites overlapping mutated regions, using various prediction tools such as the ‘Find Individual Motif Occurrences’ (FIMO). This works by scanning DNA sequences for TF binding motifs, treating each motif independently and comparing to PWMs from experimental data resources such as JASPAR [87] and HOCOMOCO [99] giving a log-likelihood ratio score for each position [100]. Other methods that are more suitable for large-scale genomic sequence data, e.g., implementing ENOCDE ChIP-seq data; tools such as the Genetic Algorithm guided formation of spaced Dyads coupled with Expectation Maximisation algorithm for Motif identification (rGADEM) [101], is a powerful approach for the discovery of de novo sequences [102]. The identification of variants that cause TF binding disruption or introduce de novo binding motif sites, offers another layer of information and significance to the regulatory effects of mutations for further experimental testing [102]. 

Despite the methods available, it is computationally challenging to predict the regulatory function of NCMs, as we lack specific gold standard tools to do so [30]. Furthermore, prediction methods are largely limited in their capacity to directly identify mutations in tumour development. However, they are a powerful tool for prioritising potential candidates for follow up functional experiments [30].

## 6. Functional and Biological Validation of NCMs

Functional validation of NCMs and regulatory affects is a fundamental step in evaluating the robustness of an in silico analysis pipeline. Multiple experimental methods can be used to demonstrate functionality. Luciferase reporter assays are the most common technique used to assess mutated enhancer regions, comparing WT and mutant sequence effects on gene expression in transiently transfected cells [30]. Reporter assays can be combined with CRISPR/Cas9 genome editing to knockin/knockout mutations from *cis*-regulatory regions. Site-directed mutagenesis and oligonucleotide synthesis can also be used to obtain the mutated sequence. Traditional reporter assays are low to medium-throughput techniques and are critical for functional validation, due to previous reports documenting inconsistencies between luciferase assay results and prediction models [62]. Several high-throughput strategies have been developed. Massively parallel reporter assays (MPRA) and self-transcribing active regulatory region sequencing (STARR-seq) in particular have been widely used [103]. Here we briefly summarise potential reporter-based methods used to validate promoter or enhancer mutated regions in Table 4.

Furthermore, ChIP-PCR techniques can also be used, particularly when validating TF-binding. However, additional assays are required after validating gene activation, to demonstrate the oncogenic properties of the noncoding mutations. For example, to determine the differences in endogenous gene expression, cell lines can undergo gene editing using CRISPR/Cas9 methods followed by quantitative PCR (qPCR) or whole transcriptome profiling [30]. Further invasion, proliferation and viability assays can be used to demonstrate the biological significance of mutations in these genome edited cell lines [30]. For example, Zhang et al., used 3D collagen hydrogel matrix models and demonstrated that NCMs resulting in the increase of *DAAM1* correlated with cell motility [20].

## 7. Conclusions and Future Challenges

Thus far, published studies have focused on driver mutations residing in the coding genome. Consequently, important therapeutic interventions to date are targeted directly towards these proteins. Somatic mutations in the noncoding genome are currently reserved for research purposes [15]. However, regulatory regions are significantly correlated with the expression of protein coding genes, warranting their importance for investigation in terms of tumourigenesis and novel biomarkers. In this review we focus on the identification of driver or important somatic mutations within *cis*-regulatory regions of the noncoding genome using high-throughput sequencing. We also discuss the in silico methods of analysis and the challenges faced. We believe integrating targeted high-throughput approaches to filtering WGS SSM data is the most efficient method of identifying and prioritising functional noncoding mutations with important regulatory effects in cancer.

Genome sequencing has revolutionised cancer studies to date [106]. With the rapid evolution of this field, and the development and improvement of chromosome capture technologies, the accuracy of linking *cis*-regulatory regions with their target genes is quickly unravelling. Somatic mutations identified within these elements can then be systematically and functionally tested in silico and experimentally. Also, further network studies such as those undertaken by Zhang et al., will provide a more integrated understanding of *cis*-regulatory associated mutations and their downstream implications [20]. This would result in the identification of important mutations and fast forward novel therapeutics to target the noncoding genome.

## Figures and Tables

**Table 1:** Noncoding mutation studies across cancer types.

Cancer Subtype	Study Source	Samples	Targeted Seq	WGS	WES	EXP	Chromatin Capture	ChIP-Seq	DNase-Seq	SNP-Arrays	ChIA-PET	FAIRE-Seq	Copy Number	Clinical Data	Resource	Identifier	Mutated Regions
**Melanoma**	Horn et al., 2013, Science.	169 cell lines 77 primary melanoma tumours	√												-	-	Promoter
**Melanoma**	Shain, et al 2015, Nature Genetics.	20 desmoplastic melanomas + matched normal samples	√	√	√	√							√		Exome and targeted sequencing: Raw Microarray data	dbGaP Accession: phs000977.v1.p1. GEO: GSE55150	Promoter
**Breast**	Rheinbay et al., 2017, Nature.	360 primary breast cancer patients + normal	√	√	√									√	Sequencing data:	dbGAP Accession: phs001250.v1.p1. TCGA	Promoter
**Breast**	Nik-Zainal et al., 2016, Nature.	560 breast cancer patients		√										√	Raw sequencing data:	EGA: Accession EGAS00001001178	Promoter
**PDAC**	Feigin et al., 2017, EMBO.	308 PDAC patients		√		√								√	WGS, Expression array, and clinical data:	ICGC AU datasets release 18 (Feb2015)	Promoter
**T-ALL**	Mansour et al., 2014,	2 cell lines, 8 T-ALL patients	√			√		√							ChIp-seq data	GEO: GSE59657	Super-Enhancer
**T-ALL**	Science. Hu et al, 2017, Blood.	31 T-ALL patients		√		√		√							Sequencing data:	EGA Accession: EGAS00001001858 EGAS00001002172	Intronic, Enhancer, Promoter
**CLL**	Puente, et al., 2015, Nature.	452 CLL patients + 54 MBL		√	√	√	√		√	√			√	√	Sequencing, expression and genotyping array data:	EGA Accession: EGAS00000000092	UTR, Enhancer
**Colorectal**	Orlando et al., 2018, Nature Genetics.	19,023 promoter fragments from cell lines		√		√	√	√					√		Hi-C, CHi-C, ChIP-seq sequencing: TF ChIP-seq: Survival data:	EGA: EGAS00001001946 GEO: GSE49402 GEO: GSE33113, GSE39582	Enhancer
**B-cell Lymphoma**	Koues et al., 2015, Cell	Purified malignant B-cells from 18 FL patients				√		√				√			All data: RNA-seq, Array, ChIP and FAIRE-seq:	NCBI Gene Expression Omnibus: GSE62246	Enhancer
**DLBCL**	Arthur et al, 2018, Nature Comm	153 DLBCL tumour/norm pairs	√	√	√	√							√	√	146 WGS sequence data: 1001 WES sequence validation data:	EGA: Accession EGAS00001002936 EGAS00001002606	3’UTR
**Liver**	Fujimoto et al., 2016, Nature Genetics	300 Liver Cancer Patients		√		√					√				Sequencing data: Mutation data:	EGA. Accession: EGAD00001001881, EGAD00001001880, EGAS00001000671, ICGC database release 18 (Feb 2015)	Promoter/Enhancer

Notes: List of noncoding mutation studies across cancer types, as well as the samples and high-throughput techniques used. Single nucleotide polymorphism array (SNP-array). Diffuse large B cell lymphoma (DLBCL). Pancreatic ductal adenocarcinoma (PDAC). T cell acute lymphoblastic leukaemia (T-ALL).

**Table 2:** List of high-throughput techniques, their functions and corresponding pros and caveats.

High-throughput Technology	Function	Pros	Caveats	Ref
**WGS**	Identify mutations genome wide	The ability to identify NCMs in all regions (not only regulatory regions). The potential identification of novel mutations implicated in cancer.	Accuracy relies on sequencing depth The alignment of short reads across repetitive regions. Large volume of data to process.	[63,70]
**WES**	Identify mutations within exon regions.	Cheaper method of sequencing the protein-coding regions of the genome. Well optimised for the identification of SNVs. ∙	Can be limited to exonic regions. Coverage is not as uniform as WGS.	[70]
**ChIP-seq**	Targeted approach to identify NCMs in putative functional regulatory regions.	Can identify putative active and repressed regulatory regions. Can be used for the identification of TF binding. ∙	Only a snap shot in time of global chromatin accessibility, which continually changes. Requires large amounts of tissue to obtain purified cells. Technically challenging to carry out.	[71]
**DNase-seq**	The identification of DNase I hypersensitivity site, mapping open chromatic genome wide.	No prior knowledge of histone modifications or TFBS need to be known.	Requires a large number of cells. Requires further ChIP analysis or functional assay to determine the function of the regulatory region identified.	[72,73]
**ATAC-seq**	Mapping chromatin accessibility genome-wide using a Tn5 transposase which inserts adaptors into regions of open chromatin	Quick processing method	Results are sensitive to variations in cell numbers.	[74,75]
**FAIRE-seq**	Allows the identification of nucleosome depleted regions, mapping regions of open chromatin.	Able to detect chromatin accessibility in a relatively low number of cells. Cheap and easy method to perform. ∙	High background noise levels, making data interpretation computationally challenging. Results are dependent on fixation efficiency.	[75,76]
**RNA-seq**	Measure of gene expression.	Can be used to identify allele specific imbalance.	Requires high read coverage to detect AI. ∙	[77,78]
**4C-seq**	Identification of long-range DNA contacts with a single genomic locus of interest.	Highly reproducible data. Ideal for analysing a known loci of interest.	Local interactions will be missed from the region of interest. Unable to detect interactions on a global level. Requires a large number of cells.	[79]
**Hi-C-seq**	Identification of long-range chromatin interactions on a global level.	An unbiased method Ideal for looking at changes within TAD regions and supra-TAD chromatin organisation.	Low resolution can be prone to high levels of noise. Requires a large number of cells. Not ideal for the identification of individual loci.	[31]
**ChIA-PET**	A combination of ChIP and 3C techniques allowing the analysis of both protein-DNA complexes and long-range interactions, genome wide.	Identifies both the DNA and protein present at a given loci.	Limited by the specificity and purity of the antibodies used.	[31,61]

Note: Transcription factor binding sites (TBFS).

**Table 3:** List of computational resources and software to identify functional noncoding variants and mutations in regulatory regions, prioritise NCMs and predict mutation enrichment in comparison to background mutational burden.

Computational Analysis Methods	Resources/Software	Method	References
**Regulatory annotation resources**	ENCODE	ChIP-seq, DNase-seq, ATAC-seq, Hi-C	[10]
	Roadmap Epigenomics	ChIP-seq, DNA Methylation, RNA-seq	[11]
	FANTOM Consortium	CAGE	[12]
**Functional Scoring**	CADD	Machine-learning algorithm	[88]
	GWAVA		[89]
	FATHMM-MKL		[90]
	Genomiser		[91]
	DeepSEA	Directly learn sequence codes from ENCODE annotations	[92]
	DelaSVM		[93]
	FitCons	Selective pressure and divergence	[94]
	LINSIGHT		[95]
	FunSeq2	Weighted scoring system	[48]
	Eigen		[96]
	IW-scoring		[83]
	Regulome DB	Heuristic Scoring	[35]
**Rate based methods with incorporated background mutation analysis**	MutSigNC		[46]
	LARVA		[97]

Note: Cap analysis of gene expression (CAGE).

**Table 4:** Gene reporter-based assays.

Traditional Reporter Based Assay	Source of DNA	Size of Test DNA Fragment	Analysis	Detection Method
**Luciferase/GFP based reporter assays**	DNA template from arbitrary source to amplify with designed primers	~1.5–2 kb	Enhancer + promoter	Luciferase activity (luminator) or GFP activity (quantitative cytometry)
**High-throughput reporter assays**				
**MPRA CRE-seq**	Microarray synthesis of DNA sequences	200–300 bp	Enhancer + promoter	RNA-sequencing
**STARR-seq**	Sheared DNA from arbitrary sources	1–1.5 kb	Enhancer discovery (also including intergenic and intronic regions)	RNA-sequencing

Notes: Traditional reporter based arrays [104]. High-throughput reporter assays [105]. Green fluorescent protein (GFP).
